# Challenges and opportunities in achieving sustainable development goal 3 in KwaZulu-Natal: reflections from a research institute, South Africa

**DOI:** 10.1186/s12889-025-24306-7

**Published:** 2025-10-29

**Authors:** Rujeko Samanthia Chimukuche, Thembelihle Zuma, G. Nduku Wambua, Thabang Manyaapelo, Anita Edwards, Natsayi Chimbindi, Kingsley Orievulu, Dumsani Gumede, Nellie Myburgh, Allanise Cloete, Janet Seeley, Nothando Ngwenya

**Affiliations:** 1 Africa Health Research Institute, KwaZulu-Natal, Durban, South Africa; 2 Division of Infection & Immunity, University College London, London, WC1E 6BT UK; 3 College of Health Sciences, School of Nursing and Public Health, University of KwaZulu Natal, KwaZulu-Natal, Durban, South Africa; 4 College of Health Sciences, School of Clinical Medicine, Department of Psychiatry, University of KwaZulu Natal, KwaZulu-Natal, Durban, South Africa; 5 Centre for Africa China Studies, University of Johannesburg, Johannesburg, South Africa; 6 Department of Global Health and Development, London School of Tropical Hygiene and Medicine, London, WC1H 9SH UK

**Keywords:** SDG 3, Rural health, Well-being, Healthcare access, South africa

## Abstract

**Background:**

South Africa is committed to achieving Sustainable Development Goal 3 (SDG 3), which aims to ensure health and well-being for all. However, in rural provinces like KwaZulu-Natal, structural inequalities, socio-cultural challenges, and environmental stressors hinder progress. This study synthesises findings from qualitative research conducted at the Africa Health Research Institute (AHRI) to explore the challenges and opportunities in meeting SDG 3 targets.

**Methods:**

An integrative literature review was conducted, analysing studies from 2015 to 2024 that focused on SDG 3related topics, including HIV/AIDS, tuberculosis, maternal and child health, sexual and reproductive health, and the impact of climate change. A framework analysis approach was applied to identify common themes, opportunities and challenges to achieving SDG 3 in rural KwaZulu-Natal.

**Results and discussion:**

Key challenges to achieving SDG 3 include limited access to healthcare, socio-cultural norms that influence health-seeking behaviours, climate-related stressors, and gender disparities. Studies highlighted poor maternal immunisation uptake due to traditional beliefs, stigma-related challenges in HIV prevention, and climate-induced economic hardships affecting treatment adherence. Gendered challenges were prominent, with men’s healthcare engagement being hindered by masculinity norms and adolescent girls facing restricted access to sexual health services. The COVID-19 pandemic further disrupted access to healthcare, particularly for older adults. Despite these challenges, opportunities exist for progress. Community-driven interventions such as DREAMS and MTV-Shuga improved adolescent engagement with sexual health education. Male-focused interventions like Stepping Stones and Creating Futures increased men’s involvement in HIV care. Additionally, integration of climate adaptation strategies into health systems could mitigate environmental health risks.

**Conclusion:**

This study provides critical insights for policymakers to enhance the progress towards achieving SDG 3. To do so policymakers should focus on addressing systemic healthcare challenges, integrating gender-responsive interventions, and strengthening community-based health initiatives. Climate-resilient healthcare infrastructure and policies are crucial to ensuring sustained progress. Future efforts should focus on expanding youth-friendly services, enhancing male engagement in healthcare, and leveraging local partnerships to improve health outcomes in rural communities.

## Introduction

South Africa signed up to the Sustainable Development Goals (SDGs) proposed by the United Nations in 2015 as part of a multisectoral approach to monitor the progress and promotion of health and wellbeing for everyone at all ages by 2030 [1]. The intention behind SDG 3 is to improve global health outcomes and address a range of health-related challenges by promoting access to healthcare, reducing the prevalence of diseases, and ensuring universal health coverage, with a focus on regions with the highest disease burden and neglected populations as priority areas [1]. Specifically, some of the SDG 3 targets aim to reduce maternal and child mortality (SDG 3.1 and 3.2), eradicate epidemics (SDG 3.3), ensure universal access to healthcare and reproductive services (SDG 3.7) including family planning, information and education, and the integration of reproductive health into national strategies and programmes. Furthermore, SDG Target 3.b, aims to support the research and development of vaccines and medicines for the communicable and non-communicable diseases that primarily affect developing countries.

The 2023 South African SDG Indicator Tracking Report [2] reports that there has been a notable decline in new infections of HIV among 15-to 24-year-olds between 2008 and 2017 from 2,3% to 1,0% (per 1000 uninfected population). The decline is attributed to the sustained efforts through the policies and programmes aimed at providing access to ART. The progress report also noted that the percentage of people living with HIV between the ages of 15 and 49 years on ART was 60,4% in 2017, with 56,3% being males. The Tracking report admits that there is still much work to be done about knowledge of HIV/AIDS for the adolescent population as 64.0% of this population did not have adequate information about HIV/AIDS [2].

To further contextualise the status of South Africa’s progress towards the targets and to highlight the need for continued efforts and innovative approaches the report indicates that the incidence of Tuberculosis (TB) per 100,000 population has progressed since the total numbers were 834 in 2015, 567 in 2017 and 554 in 2020. Furthermore, South Africa is reported to have made notable strides under SDG Target 3.b, specifically in improving access to essential medicines. From 2013 to 2022, the proportion of health facilities equipped with a core set of affordable, essential medicines rose from 74.2 to 88.6% [2]. Finally, SDG Target 3.d, focuses on enhancing national capacity for early warning and response to health risks. The reported capacity and health emergency preparedness in 2021 was 40% for coordination, 47% for communication and 60% for response. The food safety preparedness was reported as 80% in 2021 [2].

The background discussed above highlights the need for continued efforts for improvements in the progress towards the SDGs. Access to health services in rural areas is known to be more challenging than in urban areas due to geographic isolation [3]. Reports from rural health practitioners about shortages in staffing with high vacancies for doctors and nurses and reports of infrastructure challenges from unreliable electricity, water, ambulance services, and medicine stock outs are often in the news [4, 5]. The Minister of Health admitted during a meeting of the G20 planning group in March 2025 that progress towards universal health coverage was delayed [6].

This justification for this paper lies in the opportunity to use a different lens based on rigorous, mainly qualitative, scientific research conducted in a rural context, which provides information that if recognised and implemented may offer positive outcomes in the progress towards SDG 3. The paper provides a synthesis and analysis of the findings from SDG 3-related research conducted in the province of KwaZulu-Natal at the Africa Health Research Institute (AHRI) since the adoption of the SDGs, serving as a proxy for the challenges and opportunities to South Africa’s national progress towards achieving SDG 3. In many cases quantitative measures are used to indicate success in achieving these SDG targets across countries and communities [64]. Although quantitative data provides an improved understanding of disease, health and healthcare, and the success of targeted implemented programmes, they do not always offer an insight into the perspectives and interpretations of health, including the perceptions of and utility of these programmes for improving health and well-being. Qualitative data on progress toward SDG 3 can provide a more comprehensive picture of the health of a population than quantitative indicators alone, particularly in rural low-middle-income countries (LMIC), where there may be a lack of understanding about decision-making and communication processes that facilitate health and well-being [8]. They can help identify areas where progress is being made and where further improvement is needed. Reviews done on assessing SDG progress have stated that qualitative approaches might be more attractive to decision-makers and have a greater impact on national level implementation [9]. The objective of this paper is to collectively reflect on the qualitative research findings from studies carried out at a South African research institute over the past two decades with a view to both highlight the challenges that need to be overcome if progress is to be made, as well as identifying the opportunities for South Africa to leverage progress towards the SDG 3.

## Setting

### National context

The healthcare landscape of South Africa, a country that struggles with disparities in access to services, a high burden of diseases, and the ongoing impact of socioeconomic factors on health outcomes. South Africa contends with one of the highest HIV/AIDS prevalence rates globally at 12.7% [10]. Despite strides made in increasing the availability and accessibility of antiretroviral therapy (ART), challenges persist in HIV prevention, treatment adherence, and addressing socio-economic factors fuelling the epidemic. TB remains a formidable public health concern, with South Africa reporting one of the highest TB incidence rates worldwide, alongside a growing threat of drug-resistant TB [11]. Additionally, the burden of non-communicable diseases (NCDs) is on the rise, propelled by lifestyle factors such as poor diet and physical inactivity [12]. While maternal and child health indicators have seen improvements, challenges continue concerning maternal mortality rates, access to quality antenatal and postnatal care, and child malnutrition [13–15]. Healthcare access remains inequitable, with rural areas disproportionally affected by infrastructural deficiencies and workforce shortages. Mental health emerges as a burgeoning public health issue, compounded by stigma, resource limitations, and a shortage of mental health professionals [16]. Furthermore, the distribution of healthcare workers poses a challenge, with urban areas benefiting from better access compared to underserved rural regions such as rural KwaZulu-Natal [17].

Evidence suggests that the SDG goals and targets present implementation challenges, which vary depending on a country’s current state, differing responsibilities, available resources, and capacity to meet the targets [18, 19]. Owing to the persistence of social problems facing LMICs, such as poverty, health, education, and gender issues, most countries are not on track to meet many of the SDGs [7]. Significant work remains to address the socio-cultural complex problems that continue to serve as persistent challenges to achieving ‘healthy lives and well-being for all ages (SDG 3)' [20].

As a first step towards progress and as a backdrop to the challenges and opportunities to the research analysed in this paper, South Africa passed the National Health Insurance (NHI) policy in 2017 with a focus on providing access to quality healthcare services to all citizens, delivered equitably, affordably, efficiently, effectively, and appropriately [21]. The NHI Bill was signed into law on 15 May 2024 [22]. The NHI is aligned with SDG 3 and indicates South Africa’s commitment to ensuring that people’s healthcare is prioritised over the financial risk of affordability. The NHI promises to improve healthcare access and has been shown to do so in other LMIC settings with increased utilisation of facilities [23]. Despite this commitment, a significant challenge remains in implementing the NHI policies due to limited public resources, few specialist healthcare professionals, stockouts in clinics, long distances to travel to access healthcare and many other challenges reported in the literature [24–26].

The challenges mentioned above in implementing universal healthcare for all and achieving SDG 3 have been compounded in recent years by a surge in climate stress. Rural areas of LMICs are the most severely affected by these challenges. For example, in the rural areas of the province of KwaZulu-Natal in South Africa, extreme weather events have primarily manifested as droughts and flooding, posing a threat to food security and access to healthcare [26, 27]. Additionally, the COVID-19 pandemic accentuated the challenges faced in achieving universal healthcare and SDG 3 more generally. There were interruptions in the critical components of healthcare service delivery in primary health clinics, notably HIV care, TB care, HIV testing, ART initiation, and retention in care, which were measured using ART collection visits and missed patient visits [28]. Political unrest culminating in looting and destruction of public and private properties also negatively impacts vaccination rollouts and disrupts access to essential healthcare services, including the collection of chronic medication by patients with TB, HIV, and diabetes [29].

### District context

The Africa Health Research Institute (AHRI), an independent research institute situated in the uMkhanyakude District Municipality located along the coast in the far north of the KwaZulu-Natal Province, South Africa, is well-positioned to consider matters relating to SDG achievements. uMkhanyakude District where AHRI conducts population-based research is predominantly rural with high levels of youth unemployment, significant HIV-burden, mental ill-health and teenage pregnancy among adolescents [30, 31]. Healthcare in the district is mainly nurse-led and provided through the public-sector fixed and mobile clinics together with community health workers as part of the National Department of Health (NDOH) primary healthcare re-engineering policy strategy [32]. The Africa Centre Demographic Information System (ACDIS) cohort was established in 2000 by AHRI. In 2017, the cohort was expanded and renamed the Population Intervention Platform (PIP). The cohort is made up of approximately 140, 000 individuals (median age = 23 years, interquartile range = 11–36) [31].

For two decades social science and clinical research, employing a diverse array of methodologies, have been conducted in this setting providing a foundation for evaluating SDG 3 progress and synthesising the findings of research conducted by multidisciplinary scientists. A synthesis and analysis of the research studies offers insights into the opportunities and challenges for achieving the SDG3 goals in an under-resourced context.

## Methodology

### Research design

An integrative literature review [33, 34] research design was used to examine and synthesise the literature from studies conducted by AHRI in collaboration with local and international partners.

### Data collection

A literature search in the AHRI publications repository was conducted to identify the published results of studies during the period since the adoption of the SDGs by South Africa [35].

The inclusion criteria were:

- Peer reviewed articles published from 2015 to 2024.
- Studies that were qualitative in nature.
- Studies addressed SGD 3.3 and 3.7 with topics around HIV/AIDS, TB, malaria, tropical diseases, hepatitis, water-borne diseases, communicable diseases, sexual and reproductive health (SRH) and wellbeing.

Each of the studies were peer reviewed and published previously. All participant selection, data collection and ethical clearance for each of the studies was reported in the peer reviewed publications identified and will therefore not be reported here.

### Data analysis

**Fig. 1:**
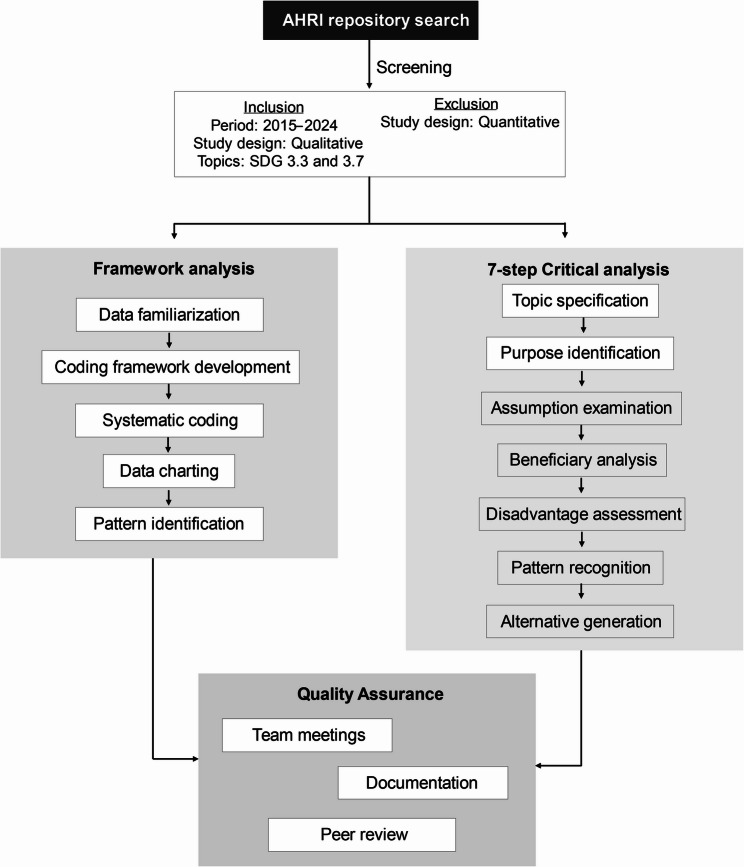
Diagrammatic representation of analysis methodology

We employed the framework analysis [36] methodology, supplemented by the seven-step critical analysis framework [37], to analyse the findings of the studies conducted at AHRI. The combined methods which we implemented are visually represented in Fig. 1. The framework analysis involves several key steps: familiarising with the published data and checking which SDG target it addresses [36].

Framework analysis is a qualitative research method for analysing qualitative data. It involves systematically applying a set of predetermined categories or codes to the data to identify patterns and themes [36]. The categories or codes are typically developed based on the research question or objectives, and can be derived from theory, previous research, or the data itself. Framework analysis is a flexible method for analysing a wide range of data types, including interviews, observations, documents and images. It allows for the systematic and efficient identification of patterns and themes. The analysis also incorporated the first two steps from the seven-step critical analysis framework: (1) name the specific topic being analysed, (2) identify the intended purpose of this analysis [37].

We developed a coding framework based on predetermined categories, coding the data according to these categories, charting the coded data, and interpreting the data to identify patterns and themes. This approach enabled a systematic analysis of the opportunities and challenges in achieving SDG targets across various datasets. Additionally, the modified critical analysis framework facilitated a deeper understanding of the topic and helped describe the studies and societal patterns. Overall, this combined methodological approach enabled us to comprehensively analyse and collate the qualitative data, providing valuable insights into this research topic.

## Results and discussion

We identified 10 studies conducted by AHRI that met the inclusion criteria. Table 1 summarises the aims of each of the studies and provides an overview of the participants in each study. The summary highlights the specific context of the various studies and offers a lens into the relevance of the findings to SDG 3. The integrative review of the ten studies identified the challenges and opportunities for progress towards SDG 3 in a rural context identified by the studies which are summarized in Table 2.

**Table 1:** Summary of included studies

#	Year	Authors	Study title (abbreviation)	Study aims	Participants	Key Findings
1.	2022	Chimukuche et al., [24]	Assessing Community Acceptance of Maternal Immunisation in Rural KwaZulu-Natal, South Africa: A Qualitative Investigation. (IMPRINT)	To assess the factors that influence pregnant women’s decision to engage with maternal immunisation	Pregnant women, their caregivers, healthcare personnel specializing in advanced midwifery and childbirth assistants, traditional medicine practitioners, religious leaders,	The findings revealed that traditional customs and institutional barriers significantly impact immunisation uptake. Key barriers included low-quality health service delivery, long queues, distance to health facilities, vaccine stockouts, and low levels of maternal knowledge. Traditional beliefs were particularly influential, with participants noting that ancestors were perceived as providing better protection than immunisation programmes. Partners of pregnant women often negatively influenced engagement with maternal care, especially when women were financially dependent on them. The study found that traditional healers encouraged clinic attendance for antenatal care but simultaneously promoted traditional medicine use, creating competing health-seeking behaviors. Water access challenges, hygiene and sanitation issues, and transportation costs further complicated access to maternal health services. The research highlighted how contextual vulnerabilities linked to poverty and unemployment exacerbated these challenges.
2.	2022	Chimukuche et al., [38]	Examining oral pre-exposure prophylaxis (PrEP) literacy among participants in an HIV vaccine trial preparedness cohort study. (PrEPVACC)	To understand factors of PrEP literacy that influence the acceptability and uptake of the HIV vaccine	18- to 45-year-olds at high risk of HIV acquisition	The study found that PrEP awareness was strongly influenced by external factors such as social media and local community knowledge about HIV treatment and prevention. However, participants reported low PrEP literacy in their communities, attributed to limited exposure to information and lack of PrEP champions in their areas. Most participants indicated that their PrEP knowledge came from joining the HIV vaccine trial preparedness study, with little prior exposure to PrEP information. The study also revealed concerns about drug effectiveness and side effects among participants. At the community level, PrEP was associated with being sexually active, leading to potential stigma. Participants noted that healthcare workers’ attitudes toward young people seeking PrEP were not always supportive, with some feeling judged when requesting PrEP at health facilities. Additionally, participants emphasized the importance of internet and social media as information sources, though participants highlighted the prevalence of misinformation. Individual and interpersonal factors were identified as barriers to accessing healthcare services, with insufficient PrEP knowledge circulation in communities contributing to low literacy levels.
3.	2022	Orievulu et al., [39]	Economic, social and demographic impacts of drought on treatment adherence among people living with HIV in rural South Africa: A qualitative analysis.	To examine the economic, social and demographic impacts of drought on health for rural people living with HIV	People living with HIV	The study found that drought-enforced soil water depletion, dried rivers, and dams created a cascade of challenges including livestock losses, reduced agricultural production, and insufficient access to water and food. These conditions indirectly impacted HIV treatment adherence through multiple pathways. Economic impacts included disrupted income and livelihoods, with participants reporting cattle deaths and crop failures that forced difficult decisions about resource allocation. The drought forced people to choose between buying food, water, and maintaining their livestock versus accessing healthcare. Social impacts included water insecurity, sanitation challenges, and health risks from using contaminated water sources. Some participants reported missing clinic appointments due to searching for water or lacking sufficient water to take medications properly. The study also revealed how climate-related stressors exacerbated existing vulnerabilities linked to poverty and unemployment, creating competing priorities that challenged HIV care utilization. Migration and livestock relocation were identified as coping mechanisms that further complicated treatment adherence.
4.	2022	Zuma et al., [40]	A socio-ecological approach to understanding experiences and perceptions of a multilevel HIV prevention intervention: The determined, resilient, empowered, AIDS-free, mentored, and safe (DREAMS) partnership in uMkhanyakude, KwaZulu-Natal, South Africa	To understand participants’ experiences and perceptions of the DREAMS intervention components	Adolescents and young people, government stakeholders and DREAMS implementing partners	The study found that while PrEP awareness among AGYW increased from 2–9% over the study period, actual uptake remained extremely low, with none of the 21 young women who sell sex (YWSS) who were aware of PrEP were using it, despite 13.4% of sexually active AGYW reporting transactional sex. The intervention showed positive effects on individual-level access to sexual and reproductive health services, but restrictive gender norms significantly limited engagement, particularly for AGYW accessing services publicly who feared community gossip and judgment. At the interpersonal level, there was limited family engagement in DREAMS programming due to barriers including transport costs and lack of parental endorsement, while organizational challenges included difficulties coordinating multiple implementing partners and limited sustainability of new programs like PrEP when DREAMS funding ended.
5.	2022	Kyegombe et al., [41]	A qualitative exploration of the salience of MTV-Shuga, an edutainment programme, and adolescents’ engagement with sexual and reproductive health information in rural KwaZulu-Natal, South Africa.	Examines the extent to which the MTV-SHUGA edutainment programme influenced young people’s engagement with sexual and reproductive health (SRH) information	Young people between 15 and 30 years who had viewed the MTV-SHUGA programme in the community viewing session	The study revealed that most participants had not watched MTV-Shuga on television due to various barriers including preference for other programmes, competition for TV time, late screening hours, lack of cable access, or discomfort watching with adults present. However, young people found MTV-Shuga both educational and entertaining when they did watch it, particularly valuing its focus on discussion and open debate about sexual and reproductive health topics rather than didactic instruction. Participants described the programme as providing an entertaining guide for navigating sexual health risks in ways that resonated with them, learning about delayed sexual debut, resisting peer pressure, avoiding intergenerational sex, and understanding alcohol’s role in compromising consent. The study highlighted the importance of enabling young people to watch MTV-Shuga with peers in safe spaces with facilitated discussion, and suggested value in encouraging parents to watch as a means of opening family communication about sexual health.
6.	2022	Chimbindi et al., [42]	(AMETHIST) Antiretroviral therapy based HIV prevention targeting young women who sell sex: A mixed method approach to understand the implementation of PrEP in a rural area of KwaZulu-Natal, South Africa.	To understand community norms and PrEP uptake amongst young women who sell sex (YWSS)	Adolescent Girls and Young Women (AGYW) aged 13–22; older community members; key informants amongst teachers and healthcare providers; 15–24-year-olds in the community	The study found that while PrEP awareness increased from 2–9% among all AGYW, uptake remained extremely low even among the target population, with only 10.6% of the 194 YWSS eligible for PrEP aware of it and none actually using it, despite 85.6% knowing their HIV status and 68.1% being HIV negative. The narrow, targeted approach significantly limited reach and accessibility, with community gatekeepers initially resisting association with sex-worker programmes and tight age/gender eligibility criteria (18–24 years) excluding many potential beneficiaries. Youth were enthusiastic about PrEP as an HIV prevention tool, while older community members were cautious about the unfamiliar technology, and teachers and healthcare providers worried PrEP would reduce personal responsibility for sexual health. The study concluded that introducing PrEP as part of broader sexual healthcare rather than through narrow targeting might improve demand and access for YWSS.
7.	2023	Gibbs et al., [43] (STEPPING STONES)	Adaptation and pre-test of a shortened Stepping Stones and Creating Futures intervention focused on HIV for young men in rural South Africa.	To adapt and test the Stepping Stones and Creating Futures programme (SSCF) for a rural context	Young men aged 18–35 years	The study found that overall uptake was 64% among approached men, with 58% attending at least one session and 71% retention among those who started. Peer recruitment was significantly more successful than random household selection with 100% uptake versus 32% respectively. Qualitative data showed the intervention was highly acceptable, with participants appreciating its discussion-based approach and focus on issues relevant to their lives rather than didactic health education they had previously experienced. The intervention supported normalization of HIV in men’s lives and recognition of health importance for achieving life goals, with six of 12 participants reporting HIV testing after the intervention. However, the study highlighted needs for greater focus on HIV-related stigma and fear, demonstrated the importance of HIV self-testing kits in encouraging testing, and revealed challenges with men using self-tests rather than accessing confirmatory clinic testing, suggesting the need for stronger linkages between community-based interventions and formal healthcare systems.
8.	2021	Adeagbo et al.,[44]	Barriers and Strategies to Improve Men’s Uptake of HIV Care Services in Rural KwaZulu-Natal, South Africa: A Qualitative Approach	To further understand the barriers and potential strategies to improve men’s uptake of HIV testing, treatment, and prevention	Males aged 18–35 years	Masculine norms, stigma, and fear of an HIV identity were identified as major barriers to HIV testing uptake among men, along with hesitancy due to perceived risks, while participants living with HIV identified various sociopsychological, structural, and COVID-related factors inhibiting the uptake of and adherence to HIV treatment. Notably, besides condoms and circumcision, no participants had prior knowledge of PrEP as a prevention method. The study found that men often delayed HIV testing until severely ill, driven by masculine pride and viewing HIV services as primarily for women, with fear of stigmatization and shame associated with HIV-positive status deterring both testing and treatment. COVID-19 lockdowns further disrupted access to HIV services through fear of infection and police harassment during travel to clinics. In response to these barriers, participants suggested that men need tailored HIV/AIDS messaging and education led by men living with HIV about the benefits of HIV testing, treatment, and prevention, and believed that community delivery of HIV services would encourage more men to engage in care.
9.	2022	Chimukuche et al., [45]	Policy and Guideline Review of Vaccine Safety for COVID-19 in Pregnant Women in Southern Africa, with a Particular Focus on South Africa	To understand the excluding and inclusion of pregnant and lactating mothers in Covid-19 vaccine trials	Not applicable	The research found that initially, in 2020, clinical and vaccine trials for COVID-19 excluded pregnant and post-partum women due to safety concerns. However, as the pandemic progressed, the World Health Organisation (WHO) acknowledged that pregnant women had significantly higher risk of severe disease and should be prioritized in vaccine trials and rollouts. The review revealed significant inconsistencies across countries in policy development and implementation. Only South Africa and Namibia had explicit policies with clear guidance on vaccination plans and implementation for pregnant women. Eight out of eleven countries analysed had no explicit policies, with little guidance on vaccination program implementation, though some countries issued official statements on social media encouraging vaccination. The study found that South Africa developed comprehensive guidelines following WHO recommendations in April 2021, with detailed messaging about vaccine safety and administration during pregnancy. The research highlighted policy-practice gaps in health responses during health emergencies and emphasized the importance of explicit, clear guidelines for communicating policy changes to vulnerable populations historically excluded from vaccine trials.
10.	2024	Manyaapelo et al.,[46]	COVID-19 and older people’s wellbeing in northern KwaZulu-Natal – the importance of relationships.	to explore the impact of the Covid-19 pandemic on the wellbeing of older people	People aged 57 years and older	The study found that having access to food, healthcare and somewhere they felt safe to stay was essential for everyone, though many participants experienced increased financial struggles as adult children who had lost employment returned home to stay. Despite these money shortages, the importance of relationships, whether familial or close community of neighbours, was consistently highlighted in participants’ accounts. Older people not only received help with day-to-day life from others, but also found solace in the company of others, with the sense of community from family and neighbours helping to ease some of the stress experienced because of the lockdowns. Using a wellbeing framework with three dimensions - material (access to resources), relational (social connections), and subjective (personal experiences) - the findings demonstrate how the importance of relationships with family and friends contributed to nurturing wellbeing for older people, showing resilience through Ubuntu philosophy of interconnectedness even during the challenging pandemic period.

**Table 2:** Summary of the opportunities and challenges to achieving SDG 3

#	Year	Title (abbreviation)	Identified challenges to achieving SDG targets	Relevant opportunities to achieving the SDG 3 target(s)
1	2022	Assessing Community Acceptance of Maternal Immunisation in Rural KwaZulu-Natal, South Africa: A Qualitative Investigation. (IMPRINT)	− Traditional norms negatively affect health behaviours preventing uptake and engagement in care e.g. perceptions that pregnant/lactating women should be protected by traditional medicine − Poor health service delivery, long queues, far distances to the health facilities, immunisation vaccine stockouts − Limited knowledge on maternal immunisation	− Tailoring of community programmes that facilitate increased vaccination uptake
2	2022	Examining oral pre-exposure prophylaxis (PrEP) literacy among participants in an HIV vaccine trial preparedness cohort study. (PrEPVACC)	− Limited awareness and knowledge of PrEP in the community. − Limited awareness of available services and their benefits − Stigma associated with HIV/AIDS and related prevention methods − Negative attitudes by healthcare providers about young people’s sexual activity both restricted their access to SRH and their willingness to seek services.	− Use of community engagement strategies for the provision of information − Tailoring information from the finding highlights to raise awareness about the benefits of PrEP
3	2022	Economic, social and demographic impacts of drought on treatment adherence among people living with HIV in rural South Africa: A qualitative analysis.	− Increased vulnerability due to drought where PLHIVs making difficult trade-offs between healthcare utilisation and pursuing economic sustenance − Drought-related effects like disruptions in income, livelihoods, and food systems imposed additional physical and mental difficulties on PLHIV impacting adherence − Water insecurity forced people to spend more money and miss clinic appointments due to walking long distances in search of water	− Adequate policies and frameworks related to planning and executing strategies that can reduce the intensity of the impact of extreme weather events
4	2022	A socio-ecological approach to understanding experiences and perceptions of a multilevel HIV prevention intervention: The determined, resilient, empowered, AIDS-free, mentored, and safe (DREAMS) partnership in uMkhanyakude, KwaZulu-Natal, South Africa	− Limited co-ordination of HIV interventions for adolescents and young people − Limited flexibility within DREAMS to respond to other health issues such as mental health and alcohol abuse − Societal norms and infrastructural factors limit AGYW access to SRHS	− Collaboration with existing organizations for rapid scale-up − Mobilisation of multiple sectors and organisations to work together to strengthen existing resources
5.	2022	A qualitative exploration of the salience of MTV-Shuga, an edutainment programme, and adolescents’ engagement with sexual and reproductive health information in rural KwaZulu-Natal, South Africa.	− Lack of adolescent-friendly services − Myths and misconceptions, stigma and discrimination, and a lack of endorsement by family and community − Negative healthcare providers’ attitudes of sexual activity younger than 18years − Limited caregiver support − Limited access to TV and internet in the rural areas	− Improving existing knowledge on SRH information and services − Adolescent-friendly SRH services in the community − Caregiver support strengthening for parents or guardians through the provision of accurate SRH information − Edutainment programmes tailored for the younger people − Safe spaces for young people
6.	2022	(AMETHIST) Antiretroviral therapy based HIV prevention targeting young women who sell sex: A mixed method approach to understand the implementation of PrEP in a rural area of KwaZulu-Natal, South Africa.	− Stigma negatively influences health seeking behaviour of female sex workers (FSW) − Negative healthcare provider interactions and lack of STI and HIV prevention services − Mobility of the FSW negatively affects their adherence to HIV treatment and SRH services − Lack of services dedicated for FSW	− Targeted and integrated community-based outreach SRH and HIV prevention services for FSW as well as young men and women who buy and sell sex within rural African settings
7.	2023	Adaptation and pre-test of a shortened Stepping Stones and Creating Futures intervention focused on HIV for young men in rural South Africa.	− Inadequate consideration of challenges related to men’s engagement in HIV prevention and treatment cascade − Poor linkage to clinics, especially for those testing HIV-negative when using self-tests − Poor feasibility e.g., online delivery of interventions, given the massive constraints of poor network access, high costs and limited access to technology	− Address gender inequalities in health systems to promote engagement by men in the HIV prevention and treatment cascade − Co-adaptation of programmes to better understand the problem of interest as well as to tailor interventions for improved effectiveness. − Increased accessibility of primary healthcare services for men which includes self-tests − Support for victims of HIV related trauma
8.	2021	Barriers and Strategies to Improve Men’s Uptake of HIV Care Services in Rural KwaZulu-Natal, South Africa: A Qualitative Approach	− Inadequate knowledge about HIV, ART, and HIV prevention methods such as PrEP and side-effects of medication − Inaccessibility of confidential HIV testing (e.g., self-testing) and inadequate access to prevention services − Resistance to knowing their HIV status and non-disclosure of status due to potential stigma and undermined masculine identity and status − Feelings of hopelessness and fatalism − Access to services due to unfriendly clinic staff, lack confidentiality, long waiting times and stigma − Food insecurity and poverty	− Improved health literacy about HIV, ART and PrEP to address myths and perceptions − Strengthen access to prevention services for men − Develop interventions that support mental health in men in particular − Enhance and improve SRH access to care for men in clinics
9.	2022	Policy and Guideline Review of Vaccine Safety for COVID-19 in Pregnant Women in Southern Africa, with a Particular Focus on South Africa	− Exclusion of pregnant women in vaccine trials leads to insufficient data on the safety and efficacy of covid − 19 vaccines for these populations − Delayed inclusion and inconsistencies in communication with the pregnant women reflected inequitable vaccine development and rollout	− Research that ensures the safety and efficacy of vaccines for populations that have been historically excluded − Clear and explicit guidelines for vaccine policies − Improve communication to ensure proper implementation of vaccination programmes
10.	2024	COVID-19 and older people’s wellbeing in northern KwaZulu-Natal – the importance of relationships.	− Social isolation due to mobility restrictions impeded older adults’ ability to engage with family and friends which negatively impacted their mental and cognitive health − Limited physical and emotional support due to restrictions on travel which prevented regular visits − Interruption of cultural practices prevented customary mourning and funeral practices which compounded grief and the psychological impact of social distancing	− Strengthening social relationships to maintain mental well-being among older adults such as promoting frequent family interactions and regular phone calls) to mitigate the impacts of social isolation − Integrate culturally grounded approaches into community health initiatives for older adults − Leveraging technology such as phone and video calls to maintain social bonds with family and friends despite mobility restrictions − Develop community-based support systems that enhance intergenerational connections to promote mental health and provide social support

The challenges and opportunities were categorized under the main topics of firstly, climate events, secondly, access to healthcare for various population groups namely pregnant mothers, adolescents, men and older people and health literacy.

### Climate events

Climate change creates unprecedented or unanticipated health problems or threats in places or populations that have not previously occurred [47]. Certain communities face more severe climate-related health impacts due to increased exposure to climate hazards, heightened sensitivity to climate stressors, existing health conditions, and inadequate resources or capacity to manage or escape these threats [48]. On the one hand, despite the availability of relevant policy and mitigation frameworks, the findings highlighted that drought exacerbated socioeconomic and demographic factors [49]. Drought-related effects, such as income, livelihood, and food systems disruptions, impose additional physical and mental difficulties on people living with HIV (PLHIV) [49]. In conclusion, the DROP-Resist study showed that the accumulation of interactive challenges from drought poses severe risks to PLHIV, including the risk of failing treatment care and HIV drug resistance [49]. These drought-related challenges have severe implications for achieving SDG target 3.3 which aims to end the AIDS epidemic by 2030. The failure to address suboptimal treatment adherence could lead to HIV drug resistance, increased infections, and mortality among the population.

### Access to healthcare for pregnant mothers

The IMPRINT study highlighted that pregnant women face challenges accessing maternal immunisation [24]. Vaccination uptake was influenced by a lack of community knowledge on immunisation, cultural beliefs and low levels of maternal knowledge subsequently influenced the choice and decision to engage with maternal immunization. These challenges negatively affected health behaviours preventing uptake and engagement in care. Challenges included poor healthcare engagement to demystify the myths, institutional challenges such as low-quality health service delivery, long queues, distance to the health facilities and immunization vaccine stockouts [24]. Both the IMPRINT and older people studies found increased need to address socio-cultural and health structural challenges is essential for achieving universal health coverage and ensuring access to quality essential healthcare services, as targeted by SDG 3 and target 3.b and target 3.5 for older people [24].

### Access to healthcare for older people

The study with older people sought to investigate the wellbeing of this group during the challenging lockdowns of the COVID-19 pandemic. The study found that older people were left vulnerable and unable to access healthcare due to limited economic resources, inadequate infrastructure tailored to older people at public health facilities and lack of psychosocial support [46]. It underscored the importance of relationships and social support systems, as well as the place of cultural practices and traditions to helping orient and locate these social relationships. The use of technology such as telephones and video calls, and community-integrated supports were identified as potential avenues to promote connection among the elderly in rural contexts.

### Access to healthcare for adolescents

Studies show that adolescents and young people continue to bear the brunt of the epidemic with failure to reach them slowing down the progress the world has made in the last two decades in tackling the AIDS epidemic [50, 51]. A 2018 UNICEF report highlights the urgent need to prioritise the health and well-being of adolescents, particularly girls, as on average, 30 adolescents aged 15 to 19 are newly infected with HIV every hour, with two-thirds being girls [50]. It was on this backdrop the DREAMS programme was implemented with the aim of halting new infections among adolescent girls and young women (AGYW) through combination approaches, and ‘packages’ of interventions [52]. In addition to DREAMS, the screening of the show MTV-Shuga was embedded to explore the potential of edutainment components to contribute to improving the sexual health of AGYW [41]. It was noted from the young people that there is a strong need for youth-friendly services for young people to receive the right information and relevant services if we are to see a reduction in new infections [53]. Scale-up was possible through multi-sector and multi-organization collaboration [42]. As with other online and media-based programmes, the work with MTV-Shuga, highlighted challenges of access to information through these modes of communication. There is, therefore, a need to tailor-make such programmes for rural contexts as well as the use of safe spaces where young people can access information and services away from routine care services due to stigma as well as the cultural and societal influences [43, 53, 54].

### Access to healthcare for men

Men are often left behind in the quest for optimal health, with the majority of programmes tailored towards women and children. This is especially the case in the HIV epidemic, where despite significant advancements in HIV-prevention and treatment, men’s engagement with these services remains a concern. A national survey showed that most men did not know their HIV status, while many of those living with HIV were not on treatment, and 40% of those on ART were not virally suppressed [55]. These two studies illuminate the challenges faced by men in seeking health services, lack of information, lack of male-specific services, fear, stigma and toxic masculine culture. The co-adaptation of the SSCF initiative saw positive engagement from men in the programme as well as acceptability of a male-specific programme geared towards ending the AIDS epidemic [43].

### Health literacy

The findings showed limited knowledge and awareness of PrEP, a significant barrier to achieving good health and wellbeing [38]. Lack of proper information impacts the individual’s knowledge of available services and their potential benefits, hindering their ability to make informed decisions about their sexual health. HIV related stigma, age-related challenges restricting young individuals’ access, and negative healthcare interactions were also found to discourage individuals from seeking SRH services such as PrEP [32, 43]. There is a need for effective communication strategies to raise awareness about PrEP and its benefits, aligning with SGD Target 3.b that aims to ensure that individuals have access to accurate information about SRH. Effective community engagement is crucial for achieving universal access to SRHs. When communities are informed about preventive strategies like PrEP, they can play a role in advocating for its availability, promoting accurate information, and reducing misinformation.

This synthesis of findings reveals key challenges and opportunities for achieving SDG 3 in KwaZulu-Natal, South Africa, reflecting broader trends across rural contexts in LMICs. Our analysis demonstrates how structural inequalities, socio-cultural challenges, and environmental difficulties converge to shape health outcomes and service delivery in rural settings. The findings highlight the complex interplay between traditional beliefs, institutional constraints, and emerging environmental challenges that collectively influence healthcare access and utilisation.

The studies consistently identified multifaceted challenges to healthcare access and utilisation in rural KwaZulu-Natal. Institutional challenges, including long waiting times, medication stockouts, and limited healthcare worker availability, continue to impede service delivery, reflecting broader healthcare system constraints in rural South Africa [56, 57]. These structural challenges are compounded by socio-cultural factors, as evidenced in the IMPRINT study [24] which highlighted how traditional beliefs and institutional challenges affect maternal immunisation uptake. Similarly, the PrEPVacc study [38] revealed how stigma and limited awareness restrict HIV prevention efforts, particularly among younger populations and key vulnerable groups [58, 59].

A crucial finding emerged regarding the impact of environmental stressors on access to healthcare and healthcare outcomes. The DROP-resistant study demonstrated how drought conditions force people living with HIV to make difficult trade-offs between healthcare engagement and basic survival needs [39]. This highlights an urgent need for climate-resilient health systems, particularly in rural areas where environmental changes disproportionately affect vulnerable populations [60, 61]. The intersection of environmental challenges with existing healthcare challenges creates compounded vulnerabilities that require innovative and integrated solutions.

Gender disparities in healthcare engagement emerged as a significant theme across multiple studies. The Stepping Stones and Creating Futures (SSCF) [43] and Missing Men studies [44, 62] demonstrated how masculine norms and stigma create challenges to men’s healthcare engagement, while DREAMS [40] and MTV-Shuga [41] highlighted challenges facing young women. These findings align with broader literature on gender-specific challenges to healthcare access in sub-Saharan Africa [63], suggesting the need for gender-responsive interventions that address both masculine norms and women’s empowerment. The success of gender-specific programming, particularly in HIV prevention and treatment, indicates promising directions for future interventions.

The results of the studies at AHRI demonstrates that successful interventions will need to address underlying structural determinants including gender norms, stigma, service delivery models, and environmental factors. Community-based interventions and differentiated service delivery have the potential to overcome these structural and socio-cultural challenges as seen in the success of programmes. This aligns with growing evidence supporting community-led health initiatives in resource-limited setting [64]. Additionally, there is need for integrated approaches that address multiple barriers simultaneously requiring coordinated efforts with multistakeholder engagement. The integration of traditional leadership structures and community health workers appears particularly effective in bridging gaps between formal healthcare systems and local communities [65].

These findings are consistent with those reported by authors in sub-Saharan Africa and specifically in South Africa [15–17].

### Recommendations for practice and research

Healthcare systems in rural KwaZulu-Natal require strengthening through integrated service delivery models including developing climate-resilient infrastructure and enhancing supply chain management to prevent stockouts to prevent suboptimal adherence to ART treatment. Further strengthening of integrated services would be the creation of youth-friendly spaces and training of specialised healthcare practitioners within existing health facilities that could help address the specific needs of young people. Additionally, expanded community health worker programmes could improve healthcare access in remote areas that is well integrated with clinic and hospital services would strengthen the integration of services for improved progress on SDG 3. Community engagement could and should be prioritised and scaled–up through the methods already used such as community health worker programmes engagement with traditional leaders and healers in health promotion. Culturally appropriate health education materials and peer-led health initiatives could help bridge knowledge gaps and address stigma will also benefit from community engagement activities. Gender-responsive programming should strive to in all planning and policy development include male-specific health services and outreach, alongside strengthened programmes addressing gender norms and health-seeking behaviours. Environmental considerations must be integrated into healthcare planning through contingency plans for service delivery during environmental crises, mobile health services for remote areas, and food security programmes for vulnerable patients. Water security measures at health facilities are particularly crucial given the impact of drought on healthcare access.

Future research should prioritise longitudinal studies tracking the impacts of interventions and expanded to include mixed-methods research approaches to better understand implementation challenges. Standardized measures for assessing SDG 3 progress in rural contexts are needed, as are studies on the cost-effectiveness of various intervention models. Key knowledge gaps include innovative approaches to overcome structural barriers, intersections between climate change and health outcomes, and the effectiveness of technology-based interventions in rural settings.

### Strengths and limitations

This synthesis benefits from a comprehensive analysis of multiple studies covering diverse populations, rich qualitative data providing deep insights into local contexts, and a strong focus on rural healthcare challenges. The integration of environmental considerations and examination of both challenges and opportunities strengthens the analysis. However, limitations include a geographic focus on one region which may limit the relevance to different contexts. Despite the strength of the methodology used in this study providing a unique lens on the challenges and opportunities to progress to the SDG 3 by focussing on the research output of a rural research institution there is a counter limitation in the potential selection bias in study participants in the studies reported on which, in turn, may have resulted a biased view and perception of the variables being investigated. Additionally, due to the qualitative nature, the studies used small sample sizes with focus on specific populations restricting the applicability and scalability of the research methods. Future studies could consider multisite studies (across countries, rural-urban contexts), implement respondent driven sampling for hard-to-reach populations and include comparative populations not exposed to interventions. A further limitation to this particular methodology is that of qualitative findings, cross-sectional in nature. This could be mitigated by a commitment to planning studies that utilize mixed method approaches, longitudinal cohort designs to track change overtime, use randomized controlled trial designs with adequate control groups where ethically feasible, with extended follow-up periods. In addition, there is need for culturally appropriate instruments, use of triangulation for mixed methods and the training of local, culturally competent interviewers to improve rapport and data quality. Lastly, the studies conducted at this research institute have a primary focus on communicable diseases such as HIV and TB with several studies exploring their intersection with maternal health, with less coverage of other health areas, which may also bias the reported challenges and opportunities for progress.

## Conclusion

Healthcare systems in rural KwaZulu-Natal require strengthening through integrated and differentiated service delivery models that reduce waiting times and improve efficiency. Community-based delivery should be prioritised through scaled-up community health worker programmes and meaningful engagement with traditional leaders and healers in health promotion. Culturally appropriate health education materials and peer-led health initiatives could help bridge knowledge gaps and address stigma. Gender-responsive programming should include male-specific health services and outreach, alongside strengthened programmes addressing gender norms and health-seeking behaviours. Additionally, the creation of youth-friendly spaces within existing health facilities could help address the specific needs of young people and expanded community health worker programmes could improve healthcare access in remote areas.

Environmental considerations must be integrated into healthcare planning through contingency plans for service delivery during environmental crises, including climate-resistant infrastructure, mobile health services for remote areas, and food security programmes for vulnerable patients. Water security measures at health facilities are particularly crucial given the impact of drought on healthcare access.

Future research should prioritize longitudinal studies tracking the impacts of interventions and expanded to include mixed-methods research approaches to better understand implementation challenges. Standardized measures for assessing SDG 3 progress in rural contexts are needed, as are studies on the cost-effectiveness of various intervention models. Key knowledge gaps include innovative approaches to overcome structural challenges, intersections between climate change and health outcomes, and the effectiveness of technology-based interventions in rural settings.

This work contributes to the growing body of evidence supporting comprehensive approaches to strengthening health system in resource-limited settings. This synthesis illustrates the intricate interplay of factors affecting SDG 3 achievement in rural South Africa. While significant challenges persist, opportunities exist for improving health outcomes through targeted interventions and system strengthening. The findings emphasise the importance of context-specific, gender-responsive, and climate-aware approaches to healthcare delivery. In conclusion, achieving SDG 3 targets will require a sustained commitment to addressing structural inequalities and gender dynamics, strengthening health systems for resilience against environmental challenges, as well as engaging local communities. 

## Data Availability

Availability of data and materials – Data sharing is not applicable to this article as no datasets were generated during the current study as a result of the review methodology used and that the data consists of the findings in published articles listed in the reference list. Each published article has a relevant statement about the availability of the data in the specific study.

## Abbreviations

AHRI: Africa Health Research Institute
ACDIS: Africa Centre Demographic Information System
AGYW: Adolescent Girls and Young Women
AMETHIST: Adapted Microplanning: Eliminating Transmissible HIV In Sex Transactions
ART: Antiretroviral therapy
DREAMS: Determined, resilient, empowered, AIDS-free, mentored and safe
DROP-resistant: Drought, Poverty and HIV Drug resistance
FSW: Female Sex Workers
IMPRINT: Immunizing Pregnant Women and Infants Network
LMIC: Low-middle-income countries
NCD: Non-communicable diseases
NDOH: National Department of Health
NIH: National Health Insurance
PLHIV: People Living with HIV
PrEP: Oral pre-exposure prophylaxis
PrEPVacc: PrEP Vaccine
SDG: Sustainable Development Goal
SSCF: Stepping Stones and Creating Futures
SRH: Sexual and reproductive health
TB: Tuberculosis
UNICEF: United Nations Children’s Fund
WHO: World Health Organisation
YWSS: Young Women who sell sex
